# Multiscale Modeling of Spheroid Tumors: Effect of Nutrient Availability on Tumor Evolution

**DOI:** 10.1021/acs.jpcb.2c08114

**Published:** 2023-04-03

**Authors:** Jakob Rosenbauer, Marco Berghoff, James A. Glazier, Alexander Schug

**Affiliations:** † Jülich Supercomputing Center, Forschungszentrum Jülich, Jülich, Nordrhein-Westfalen 52425, Germany; ‡ Steinbuch Center for Computing, Karlsruhe Institute of Technology, Karlsruhe, Baden-Württemberg 76021, Germany; § Biocomplexity Institute, Indiana University, Bloomington, Indiana 47408, United States; ∥ University of Duisburg-Essen, Duisburg, Nordrhein-Westfalen 47057, Germany

## Abstract

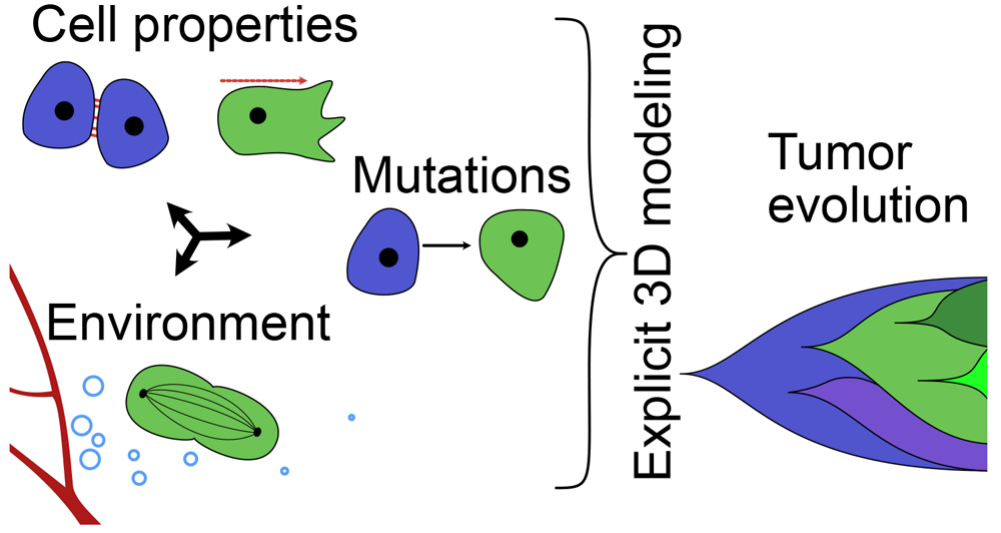

Recent years have revealed a large number of complex mechanisms and interactions that drive the development of malignant tumors. Tumor evolution is a framework that explains tumor development as a process driven by survival of the fittest, with tumor cells of different properties competing for limited available resources. To predict the evolutionary trajectory of a tumor, knowledge of how cellular properties influence the fitness of a subpopulation in the context of the microenvironment is required and is often inaccessible. Computational multiscale-modeling of tissues enables the observation of the full trajectory of each cell within the tumor environment. Here, we model a 3D spheroid tumor with subcellular resolution. The fitness of individual cells and the evolutionary behavior of the tumor are quantified and linked to cellular and environmental parameters. The fitness of cells is solely influenced by their position in the tumor, which in turn is influenced by the two variable parameters of our model: cell–cell adhesion and cell motility. We observe the influence of nutrient independence and static and dynamically changing nutrient availability on the evolutionary trajectories of heterogeneous tumors in a high-resolution computational model. Regardless of nutrient availability, we find a fitness advantage of low-adhesion cells, which are favorable for tumor invasion. We find that the introduction of nutrient-dependent cell division and death accelerates the evolutionary speed. The evolutionary speed can be increased by fluctuations in nutrients. We identify a distinct frequency domain in which the evolutionary speed increases significantly over a tumor with constant nutrient supply. The findings suggest that an unstable supply of nutrients can accelerate tumor evolution and, thus, the transition to malignancy.

## Introduction

The development of a tumor originates from healthy cells within a tissue. Through mutations and adaption to their surrounding, cells can acquire properties that enable them to grow faster than their healthy counterparts. The human body has many independent control mechanisms to ensure tissue homeostasis and to prevent the growth of tumors, such as DNA repair, cell growth and death signaling, and the immune system. For a tumor to succeed in this enviromnemt, its cells have to evade these control mechanisms. A minimal set of mechanisms that needs to be overcome by a tumor to become malignant has been identified and they are termed the hallmarks of cancer.^1^

Computational cell based modeling of tumor development and heterogeneity is emerging as a method to study hypotheses of tumor dynamics.^2^ Cell based modeling has been successfully applied. In ref (3), a cellular automaton model was used to observe the influence of cellular movement, division, and death rates on tumor heterogeneity. The use of computational models in clinical applications and personalized treatment plans is gaining momentum.^4,5^ Büscher et al.^6^ compared adhesion and “growth strength” in an evolving tumor and found that in some cases a mixture of different cell types can be the steady state instead of one cell type outgrowing others. The importance of cell–cell adhesion for tumor invasion has recently been highlighted by Ilina et al.^7^ In ref (8), the authors showed with a mathematical model using evolutionary game theory that tumor heterogeneity and the optimal cell phenotype depends on the microenvironment and position within the tumor. Cell adhesion and cell motility have been shown to strongly influence breast cancer invasion behavior and differentiate between solid-like, fluid-like, and gas-like behavior.^7^ Confinement by the extracellular matrix and cellular motility were recently investigated systematically and a phase space of tumor invasion modes was proposed that couples the invasive behavior strongly to the density of the surrounding media for spheroid tumors.^9^

The microenvironment of a developing tumor can influence the fitness of cells and therefore the tumor composition, which in turn shapes the microenvironment. A developing tumor expands in volume and requires nutrients that can only diffuse a finite distance within a tissue. Angiogenesis, the growth of new blood vessels, is initiated to sustain nutrient supply. Rapid proliferation of cancer cells leads to a volume increase of the tumor, which introduces pressure and thereby solid stresses within the tumor that can collapse blood vessels.^10^ This interplay of formation and collapse of blood vessels can lead to a fluctuating availability of nutrients for the cells in the developing tumor. During tumor development, single cell effects are of great importance, with mutations occurring initially in a single cell. Stochasticity and rare events are non-negligible during tissue development. As we showed in,^11^ such stochastic single cell effects can strongly impact tissue patterning. Previous simulation studies have investigated the evolutionary behavior of tumor models under the influence of external nutrient supply and fluctuations.^12^ The evolutionary behavior is based on the accumulation of a large number of mutation events. However, the number of cells and the spatial structure are restricted by using a 2D lattice; therefore, the mutation rates and parametric changes at each mutation have to be large. With our large scale simulation method we are capable to simulate 3D tissue with a larger number of cells for a longer time and are therefore able to reduce mutation rates.

Here, the recently developed framework CellsInSilico^13^ is used for the simulations, it is based on the cellular Potts model (cpm)^14^ that has been established for the single cell based simulation of tumor growth.^15^ Through parallelization and optimization for supercomputers, the framework enables large-scale three-dimensional simulations and a large number of replicates.

The cellular Potts model (CPM) is a lattice based model for the simulation of cellular dynamics, that has been proposed 1992 by Graner and Glazier,^14^ and it has been further developed and established as a model for the dynamical simulation of cellular mechanics. In this lattice based model, one cell occupies a set of connected lattice points. Each cell is described by a unique integer number that represents the spin in a Potts model. A Hamiltonian energy function is defined and determines the cellular behavior. In most basic cases, the energy function consists of a volume, surface, and adhesion term,^14^ but additional forces such as motility or polarization can be added. The temporal dynamics of the system are introduced by local changes of spins on the lattice. On each lattice site, a spin flip to one of the neighboring spins is attempted, and the change is accepted or rejected based on the Hamiltonian energy function and the Metropolis criterion. The system incrementally moves toward lower energies, and the local changes describe the temporal dynamics and fluctuations of the system. The parameters in the CPM Hamiltonian of this energy-based model can be mapped to physical forces.^16,17^ In the past decade the CPM has been successfully applied in modeling tumor development and invasion.^15^ Furthermore, the model was extended to include more complex surroundings such as blood vessels,^12^ cell–extracellular matrix interactions,^18^ and external signal fields in morphogenesis.^19^

Fitness is not an inherent property of the cell but a combination of the cell’s ability to proliferate and confounding factors imposed by the local environment. Modeling allows us to unravel the two influences on cellular fitness. Here, we ask how tumor evolution is influenced when only the physical location within the tumor influences cellular fitness. We introduce two variable properties of cells, namely *cell–cell adhesion* and *motility*; both can change incrementally at cell divisions with a low probability (mutation rate). In our simulation study, we distinguish between different cell types, which we call cancer and healthy cells. While healthy cells participate in the dynamical interactions of the model, they can not divide or die. Cancer cells on the other hand can divide, mutate, and die. Within the cancer cells, we distinguish between cancer phenotypes. Each cancer phenotype has a unique combination of adhesion and motility parameters. The simulated spheroid tumor consist of populations of cells of different cancer phenotypes. The proportions of the different cancer phenotypes that the spheroid is comprised of at a given time, we call the tumor composition. Division and death rates of the cancer cells are identical for all cancer phenotypes. Therefore, the fitness of a cancer phenotype is influenced by how the mechanical properties of the cells influence their environment and location.

Phenotypical changes to cells with higher motility and epithelial to mesenchymal transition are associated with tumor invasion.^20−22^ Increased motility is a landmark for the development of metastases^23^ and tumors consistently produce high motility cell phenotypes. Here, we ask whether mechanical and environmental constraints are sufficient to drive the evolution of tumor composition toward high motility. In a dynamically changing nutrient environment, cells with higher motility could dynamically adapt to the most advantageous positions; therefore, a dynamic nutrient field could induce an evolutionary advantage for motile cells. We investigate whether fluctuations in nutrient supply alter the evolutionary optimum for motile cells.

We observe an avascular tumor. The nutrient availability on the surface of avascular tumors is higher than in the center of the tumor since they interface with healthy tissue. Cancer cell phenotypes with low cell–cell adhesion can mechanically sort to the outside of tumors,^14^ providing a higher supply of nutrients and therefore greater fitness. We will test this prediction and observe whether cells expressing low adhesion have an evolutionary advantage in an evolving heterogeneous tumor. The loss of adhesion has been identified as a step toward malignancy, allowing cells to detach from the primary tumor.^24^ We thus ask whether purely geometric and mechanical constraints can induce a fitness advantage of low adhesion cell phenotypes.

We simulate a three-dimensional spheroid tumor that is surrounded by a population of healthy host tissue. During the simulations, we observe the evolution of the tumor composition along two independent parameters: cell–cell adhesion and cell motility. At each division, cancer cells can alter their phenotype, and therefore alter their parameters in incremental steps, with a small probability (4%). Parameters are changed incrementally, which implies continuous adaptation to the surrounding.

We observe three distinct cases. In the first case, cancer phenotypes compete only for the available space in the system. The space is limited, which leads to competition over space, and for one cell to divide, another cell has to die. Therefore, the fitness of a cell is determined by the local pressure and density of its surroundings.

In the second case, in addition to competition over space, cell division and cell death are nutrient dependent. We introduce a linearly decreasing supply of nutrients within the tumor. Therefore, the fitness of a cell is influenced by its local supply of nutrients, local pressure, and density.

In the third case, the supply of source nutrients is moved spatiotemporally within the simulated tissue. Therefore, cells experience fluctuating nutrient supply in their local environment.

## Methods

Energy functions making up the Hamiltonian energy function are

- Volume
- Surface
- Adhesion
- Random motility
- Central potential

### Adhesion

The cell–cell adhesion (Table 1) is proportional to the contact area between cells and independent of the duration of the adhesion. The strength is not limited or quantized by focal adhesion but is only determined by the adhesion parameter between the cell types and the shared area.

**Table 1:** Parameters Varied during Simulations

parameter	Range
cell–cell adhesion	[0···2*T*_MC_] (no repulsion) discrete values: [0, 10, 20, 30, 40, ..., 100, 110]
motility	[0···2*T*_MC_] discrete values: [0, 10, 20, 30, 40, ..., 100, 110]
Metropolis temperature *T*_MC_	55 (constant)
motility recalculation Time *t*_motility_	100 MCS
system size	200 × 200 × 200 μm ≈ voxels
coupling to central potential	–70
cell volume	500
cell volume coupling	8
cell surface	400
cell surface coupling	6

### Nutrient

The nutrient availability of a cell is determined by its location in 3D space. The position of a cell is defined as the center of mass of its spatial extent. The function is a radially linear decay within a sphere, in the center of the simulation box. The center of the nutrient well can be temporally constant or moving, to represent constant or dynamic tumor environments. The nutrient represents a growth-limiting factor for the cells.

### Central Potential

To avoid all cells accumulating in the outer regions with constant high nutrient availability, a potential is introduced. This potential leads to all tumor cells experiencing a constant force toward the center point of the simulation. This point is also the center of the nutrient well, with the lowest availability. The potential leads to an increase in pressure at the center of the tumor.

### Random Motility

Motility is implemented by assigning a preferential direction of movement to each cell. This direction is defined by a potential along a vector. The three-dimensional direction of this vector is randomly reassigned in a regular interval of 100 Monte Carlo sweeps. The cells are coupled to this potential by a constant force that is determined by the coupling of the energy term to the potential. This coupling constant varies for different cell types and is referred to as the motility strength in this manuscript.

### Cell Division and Death

To divide, cells need to exceed the age of 2 kMCS and their volumes have to exceed 90% of their goal volume. Similarly, cells can divide once they exceed the age of 4 kMCS.

There are two different cases for dependency of cell division and death on nutrient availability:

1. No dependence: Constant division probability of division and death.
2. Linear dependence.
  - Division: Rate linearly increases from 0 to ≈0.005 with increasing nutrient availability
  - Death: Rate linearly decreases from 0.001 to 0 with increasing nutrient availability

The tissue that surrounds the tumor does not participate in cell death or division. The cells that make up the surroundings, therefore, participate in the entire simulation and act as a medium that the tumor cells at the tumor edge can interact with and redistribute forces and pressure.

### Evolution Speed and Spread

The speed of evolution is calculated by tracking the center of mass of the distribution of cell types in the phenotype space. The spread is measured by the extent of the distribution.

## Results

**Figure 1:**
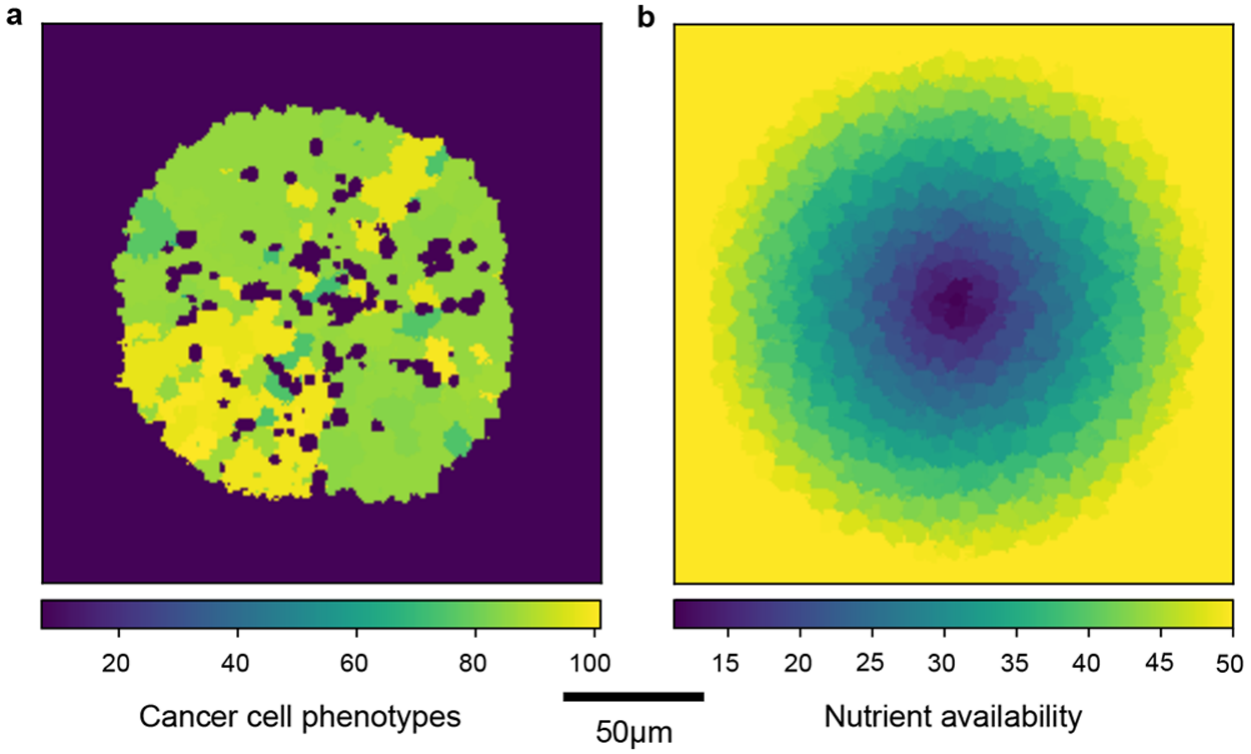
Geometry of tumor simulations. (a) Spatial organization of the cancer phenotypes within the tumor, slice through the center of the simulated three-dimensional spheroid. The cells are colored by cancer phenotype clone, and different colors do distinguish different phenotypes and do not directly indicate the parameters of the clone. Cancer phenotypes are spatially organized as wedge shaped populations, with high proliferation at the rim of the spheroid and cell death in the center. The colorbar shows 100 of 144 possible cell types; cell types above index 100 were not present in the visualized simulation. (b) Cells are colored by nutrient availability.

The simulations are performed on a three-dimensional grid of 200 × 200 × 200 voxels, here 1voxel ≈1 μm, with periodic boundary conditions. Each cell occupies a volume of *V*_0_ = 500 μm^3^ in the grid. The simulation is initialized by filling the grid with cells of the healthy cell type, they are therefore nondividing and nondying cells. In the center of the grid, a cluster of cancer cells is deposited consisting one cancer phenotype, those cells can undergo cell division and cell death. The initial cancer phenotype parameters are chosen to be in the center of the range of the variable properties, adhesion and motility. A spheroid tumor develops from this initial tumor seed of tumor cells (cf. Figure 1) in a surrounding of healthy cells. Tumor cells are subject to a potential that pulls them toward the center of the simulation grid in order to keep the tumor cells in the center of the simulation grid and prevent healthy cells to be included into the tumor. With each cell division of a cancer cell, the daughter cells have a 4% probability of changing one of the parameters, cell motility or adhesion, by a small increment or decrease. This equates to a change of the cancer phenotype of one of the daughter cells. The tumor cell division rate is larger than their death rate, therefore the number of tumor cells increases. Tumor cells can only divide when the cell volume is greater than 450 μm^3^ (0.9*V*_0_). This leads to a limit on the absolute number of cells in the system, as cells are confined in the grid and compressed as soon as the number of cells exceeds . Therefore, the tumor cells grow to a spherical tumor in the center of the simulation box. After initial expansion, tumor size remains constant while an equilibrium between cell division and cell death is reached. The effective division rate in this state is limited by the death rate. This interplay of division and death rates leads to a continuous occurrence of divisions with accompanying parameter changes in the daughter cells, and we will refer to those parameter changes as mutations. Therefore, the distribution of the parameters of the cancer phenotypes (adhesion and migration) can change over time. Here, we observe the development of this distribution over time (cf. Figure 3, top).

### Constant Environment

First, we investigate the development of the distribution of adhesion and migration parameters over time of the system as described above. Division and death of a cell are determined by the respective rates, age, and volume constraints. The tumor grows until spatial constraints limit cell division. Cell division is limited to cells that occupy a larger volume than a threshold volume (*V*_THRS_ = 0.9*V*_0_) and cells are only compressible to a finite extent. As noted above, the absolute number of tumor cells is limited. The cells thus compete over the available space, and the fitness of a cell is influenced by whether its local surrounding allows division.

**Figure 2:**
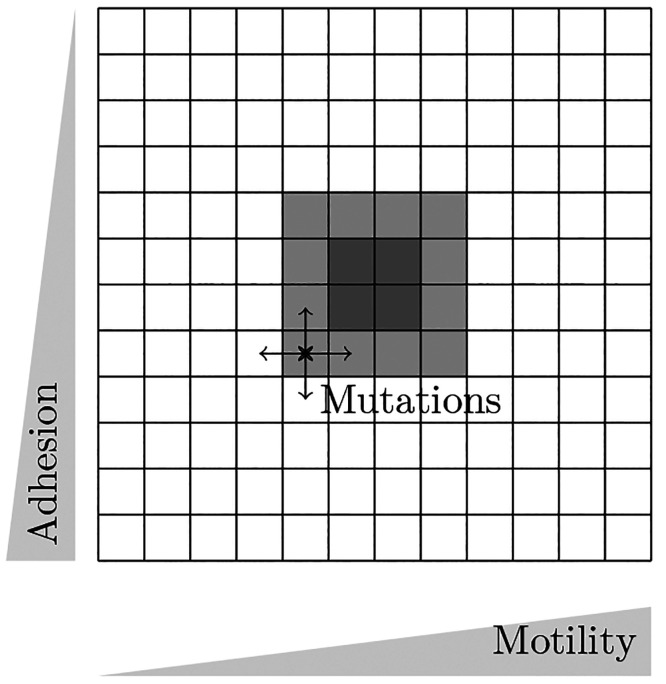
Cancer cell phenotype parametrization in a 12 × 12-matrix.

**Figure 3:**
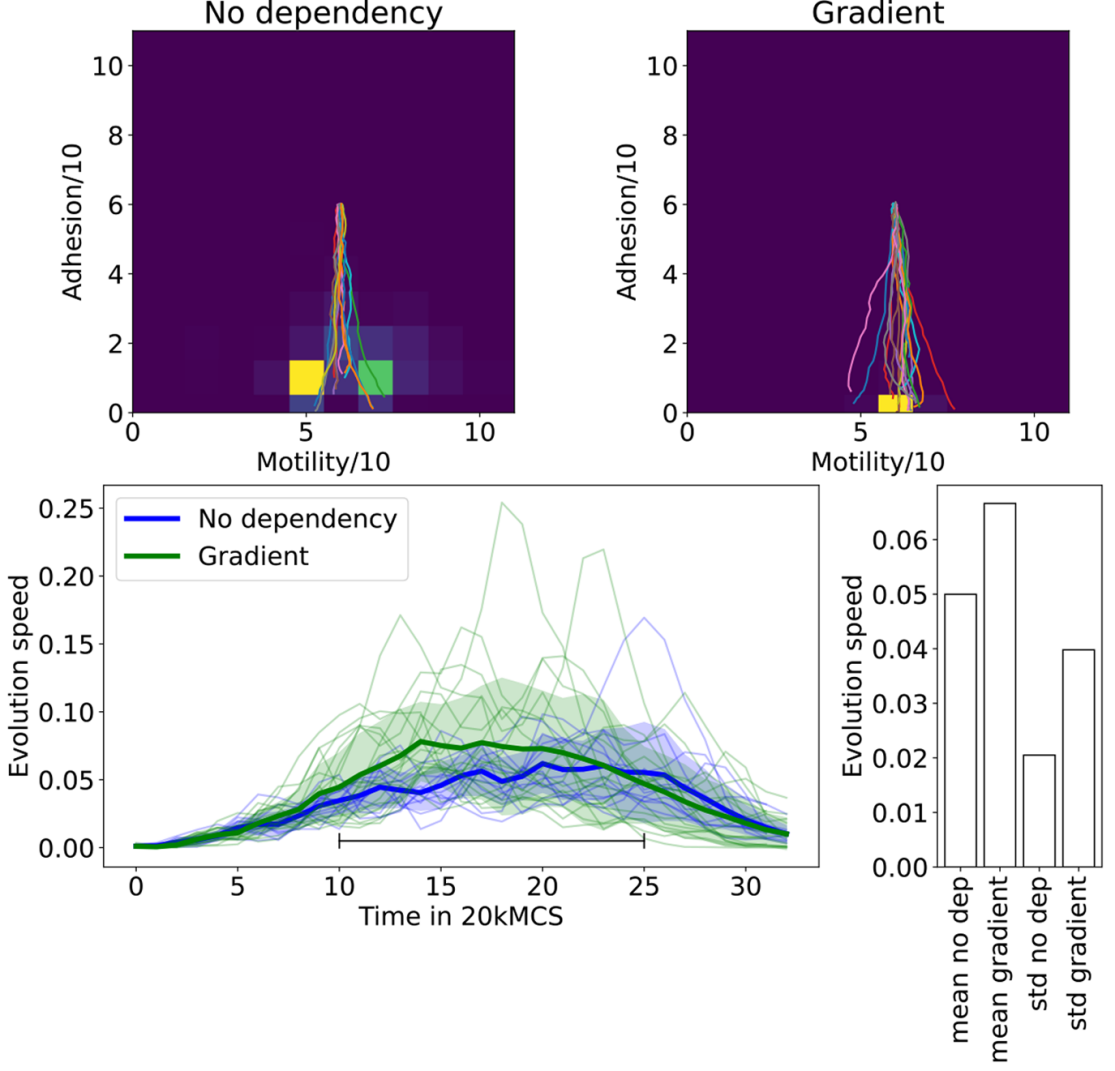
Spheroidal tumor growth for different nutrient dependency mechanisms. The top plots show the temporal trajectories of the centroid of the phase space occupation, originating in the center and developing toward low adhesion. The shading shows the distribution in the phenotype space at the end point at *t* = 580 kMCS of a single simulation, with bright colors indicating high occupation of the cancer phenotype. The bottom plot shows the evolution speed of different iterations over time, as well as the mean evolution speed and standard deviation (shaded) of this case. Two different cased of dependencies of the division and death rates of single cells are compared: no dependency of cell division and death on nutrient availability and a linear dependence of the rates on the nutrient availability. The bottom right plot compares the overall mean evolution speed and standard deviation between the constant and the gradient dependent case.

Observing the occurrence of cell events in relation to the radial distance to the tumor center, we find that cell deaths are located in the tumor center while cell divisions are located at the tumor margin. Due to mechanical constraints in the tumor center, cell death events are concentrated toward this region, while cell division is mainly occurring at the tumor border. These two processes together, lead to an overall inward movement of cancer cells toward the tumor center (as seen by the wedge shaped patches of different cancer phenotpyes in Figure 1). This behavior can also be observed in the movies that are available in the Supporting Information, there the dark blue cells indicate dying cells and cell death colocalizes with the tumor center as well as low nutrient availability. The emergence of wedge shaped regions that are primarily occupied by one cancer phenotype can be observed. There is a buildup of pressure inside the tumor, this is facilitated by the inward movement of the cells and the central potential. Through mutations accompanying cell divisions, new cancer phenotypes are introduced, leading to a distribution in the parameter space around the initial cell type. The parameter combinations of all implemented cancer phenotypes can be represented by a 12 × 12 matrix (compare Figure 2). The state of a tumor can be characterized by the distribution of cancer phenotypes in this matrix. The centroid of the distribution of this matrix is used to track the evolution of the tumor through the parameter space over the simulated time. With simulation progress, the distribution moves in the parameter space. This gradual change in the distribution is driven by phenotypes with higher fitness (i.e., producing more offspring) as the absolute number of cells remains constant. Therefore, the movement of the centroid of the distribution can be interpreted as moving toward the cancer phenotype with the highest fitness in the simulation. Furthermore, the spread of the distribution and speed of the centroid are analyzed. With the model introduced so far, we find that the tumor evolves toward the low adhesion regime, clearly visible in Figure 3. Individual trajectories start with a high directionality toward low adhesion, no directional preference along the motility axis is visible (see Figure 3, top left). Mechanical interactions and competition for space within the tumor suffice to introduce an evolutionary advantage of low adhesion cells for spatially independent division and death rates.

**Figure 4:**
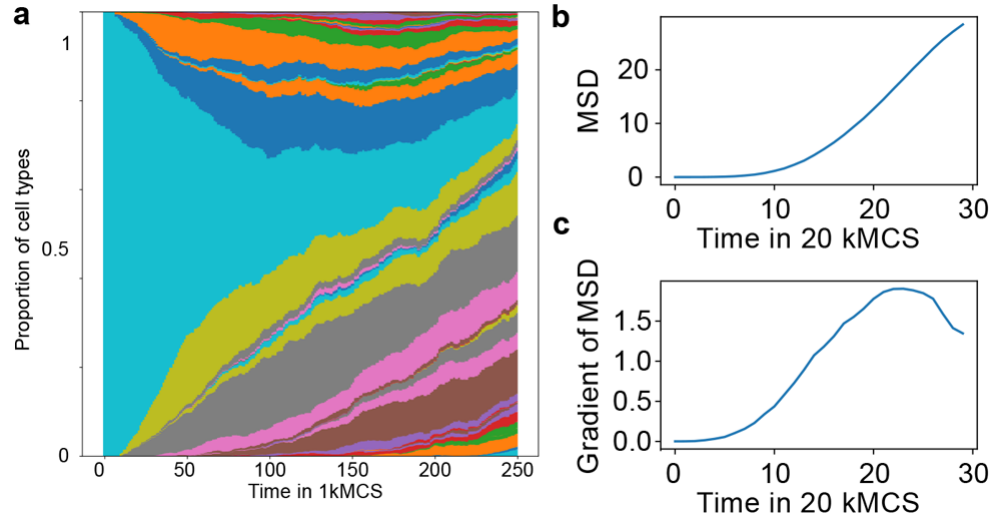
Cell type composition and mean square displacement of cell type distribution over time. (a) Relative tumor composition over time for a single simulation. The colors indicate the different parameter combinations of cancer cell phenotypes. The color coding is independent of the parameters of the phenotype, the temporal development of the parameters can be seen in Figure 3 and supplemental movies. Mean square displacement (MSD) of the distribution of parameters over time (b) and gradient (c) of MSD of 20 simulations with constant nutrient surrounding and a continuous dependency of cell division and death on the nutrient availability. The speed of the evolutionary trajectory decreases as the distribution reaches the limits of the variable parameters.

### Nutrient Dependency

Next, we investigate how nutrient dependence of cell division and death influences the development of tumor cell parameters in our model. By introducing a dependency of cell division and death on nutrient availability, cells compete over both space and nutrients. Nutrients are introduced as a growth limiting factor, representing, e.g., oxygen or glucose. High nutrient availability increases the division rate and decreases the death rate, whereas low nutrient availability decreases the division rate and increases the death rate (see Methods).

In an *in vivo* tumor, nutrients diffuse into the tissue originating from the vasculature and are gradually depleted. Here, we simplify the nutrient distribution, and it is implemented by introducing nutrient availability solely dependent on the position of the cell relative to the center of the spheroid. Cell nutrient availability increases linearly from tumor center to a maximum value and remains constant for further distances, as pictured in Figure 1b.

We introduce a dependency of the cell division rate and death rate on the nutrient availability (see also Methods). Here, cells linearly adapt the division and death rates, depending on the local concentration of nutrients. The introduction of a nutrient dependency affects the evolution speed of the tumor (see Figure 3, bottom). In the regions in which cell death and division co-occur, competition between different cell types is more pronounced, since cells that can stay in this region or escape toward the surface of the tumor will survive, while cells that are pushed to the inside of the spheroid die. The evolution is highly directional toward low adhesion for both no and linear nutrient dependency. The development is not significantly changed by the introduction of nutrient dependencies of cell death and division on the migration axis. The nutrient dependence of cell death and division leads to a shift of cell death toward the inside of the tumor and a shift of cell division toward the surface. This enhances the fitness of cells that mechanically move to the surface of a tumor (i.e., cells with low adhesion); therefore, the selection of low adhesion cells is accelerated. The mechanism of nutrient dependency shows a larger selective pressure on the tumor and therefore leads to a faster evolution. Additionally we tested a threshold-based nutrient dependency (cf. Figure S2), which significantly decreased the evolution speed. As a linear dependency is biologically more reasonable, as cells adapt proliferation rates continuously,^25^ we use the linear dependency in this manuscript.

### Dynamic Environment

To mimic the rapidly changing environmental conditions in an developing tumor we next model temporal changes of the nutrient availability within the tumor. We want to capture the dynamical environment within an *in vivo* tumor within our simplified spheroid simulation model. We introduce a periodically changing nutrient availability, as we are interested how the time scales of nutrient fluctuations and cell divisions interact and influence the evolutionary trajectory. The simplest method to introduce these fluctuations is to temporally modulate the overall availability of nutrients in a sine, or triangle pattern, adding one parameter the period *T*. However, this would restrict the frequency domains, as for long periods *T* the tumor would simply die, as cell division is suspended for low nutrient availability. Furthermore, the fluctuations on nutrient availability through angiogenesis, blood vessel collapse, and rapid expansion in *in vivo* tumors does affect local regions within a tumor and not the entire tumor at one time. This could be realized by moving localized nutrient sources, which would introduce large areas of low nutrient availability. This would again restrict the frequency domain for low frequencies, as the large regions with high death would lead to the death of all cancer cells in that region and therefore the loss of the evolutionary trajectory; future work with larger simulated areas can incorporate moving sources. Therefore, we introduced a spatiotemporal variation in the nutrient availability, by moving the negative nutrient source (which was previously in the center of the spheroid cf. Figure 1), in a circle with radius *A* = 50 around the center of the spheroid. The center of the negative source is moved in a periodic manner, while all other parameters of the simulation remain the same. The center follows a circle in the *xy* plane with amplitude *A* = 50 and period *T*. The movement of the source and the colocalization of low nutrient availability and cell death can be observed in Movies 2 and 3 in the Supporting Information.

The period *T* of the dynamics does not significantly affect the direction of the temporary development of the tumor cell parameters. The centroid of the composition systematically moves toward low adhesion, and still no significant change on the motility axis is observed. We hypothesize that the introduction of a temporally changing nutrient availability could introduce a higher fitness for cells with higher motility. We could not confirm this here.

#### Spread of Distribution

**Figure 5:**
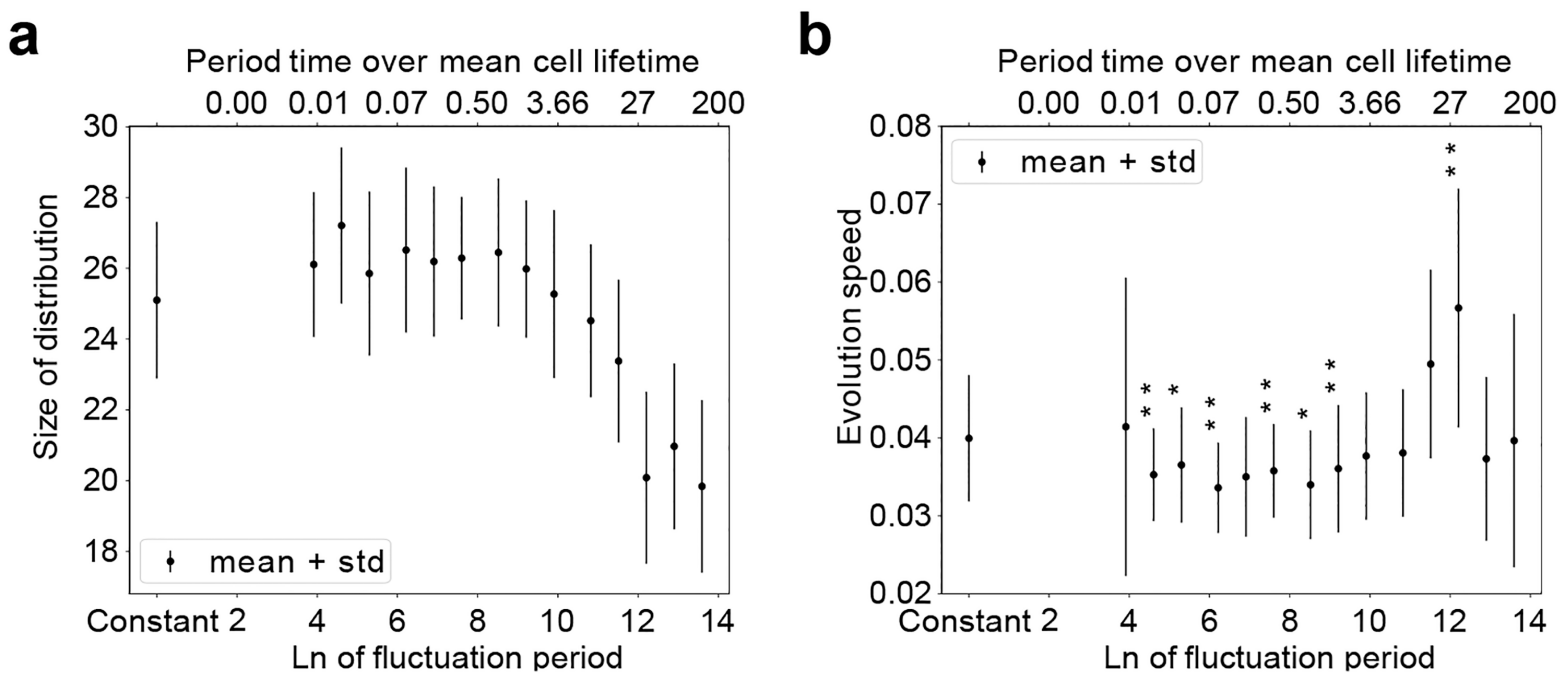
Macroscopic tumor properties for linear dependency of division rates to nutrients. (a) Width of the cell type distribution in dependence of the fluctuation period of the nutrient availability. Measured as the number of cancer phenotypes in the spheroid with more than 10 cells. Averaged over the simulation time per simulation. Depicted are the mean and standard deviation of *N* = 21 simulations. “Constant” labels the simulations with stationary nutrient availability without temporal fluctuations. (b) Evolutionary speed of centroid trajectory in phenotype space plotted against the fluctuation period *T*. Averaged across each simulations, the plot shows the mean and standard deviation of *N* = 21 simulations. A *t* test is performed for each set of simulations with a different *T*, comparing the measured outputs against the constant case. *p*-Values are depicted for all simulations with a significant impact of the fluctuations on the evolution speed (*p* < 0.04).

The spread of the distribution is measured by counting the number of parameter combinations that are occupied by more than 10 cells. We observe how this spread is influenced by the fluctuation period *T* in Figure 5a. For fast fluctuations in the availability of nutrients, an increase in spread over the constant case can be observed. With slower fluctuations, the spread first stays constant, but above *T* = 5 kMCS a clear decrease in the spread is visible. This smaller spread indicates a more directed evolution.

#### Evolution Speed

While the introduction of a fluctuating nutrient availability does not change the direction of development in the parameter space, the speed of the development is influenced as depicted in Figures 4, Figure 5b, and SI3. The mean and standard deviation of the evolution speeds of 21 replicates are shown in Figure 5b, in relation to the fluctuation period *T*. Here, a clear influence of the fluctuation period *T* on the evolution speed is visible. While the evolution speed remains at a lower level than in the constant case for short periods (*T* = 100 MCS–50k MCS), a distinct increase for *T* = 100–200 kMCS is visible with a maximum evolution speed at 200 kMCS, followed by a reduction in values to match the constant case (*T* = 0). This dependence of the maximum evolutionary speed on the availability of nutrients in the microenvironment can be seen as an emergent reaction of the tumor to its changing environment. It may enable a faster adaptation of a tumor when it is faced with an unstable surrounding and therefore accelerate its progression toward malignancy.

## Conclusions

The development of malignant tumors can be explained as a result of tumor evolution. Here, we used cell based simulations of a spheroid tumors to observe tumor evolution. To grow and spread successfully in an organism, tumors must overcome a variety of control mechanisms. Tumor evolution explains this development as a result of competition between cell populations with different properties. Cells with properties that are favorable to their fitness grow faster and therefore become more dominant in the tumor. In *in vivo* tumors, solid stresses build through tissue displacement that are capable of compressing and blocking blood vessels.^10^ This, along with angiogenesis, can lead to fluctuation in the availability of nutrients in tumors.

We present a computational model of a spheroid tumor in surrounding tissue. Mutation of cells is enabled by an incremental change of a cancer cell phenotypes parameters at cell division. Two parameters can be changed during a mutation: cell–cell adhesion and cell motility. The system is allowed to evolve freely, and the tumor composition is tracked in parameter space over time.

We find that the mechanical properties and spatial constraints of the system are sufficient to drive the ensemble toward low-adhesion cancer cell phenotypes. This mechanical effect on tissue evolution has been described in.^6^ Mechanical properties alone increase proliferation at the edge of the tumor and cell death in the center. We introduce a dependency of cell divisions and death on nutrient availability. The availability is linearly decreasing toward the tumor center. Using a linear dependence on the availability of nutrients for proliferation and an inverse linear dependence of cell death leads to higher evolutionary speed. We investigate how fluctuating nutrient availability influences tumor evolution. We find a dependency of dynamic nutrient surroundings on the evolutionary speed. The speed shows a frequency dependency, with a lower evolutionary speed for fast fluctuations followed by an increase over the constant case for slower fluctuations. There is a critical time scale for fluctuation of nutrient availability that provides a distinct peak in maximal evolution speed, which we find to be between *T* = 100 kMCS and *T* = 200 kMCS. This time range is between 15 and 30 cell generations (average lifetime of a cell ≈7 kMCS, cf. Figure S1). This behavior was not expected, and we observe an decrease and increase of evolution speed for different fluctuation periods of the nutrient availability. We hypothesize that the alternating periodic occurrence of locally high cell death and high proliferation leads to an increased selection toward low-adhesion cells. During the high proliferation phase, the local composition is expanded, cells with higher fitness grow marginally slower than other cells in their surrounding. By decimating the number of cells in this region through high cell death, phenotypes with low occurrence and low fitness are more likely to die out. During the following high-nutrient phase the growth rate is increased, as there are less phenotypes present, the directionality of the evolution can be increased. This succession of the reduction of number of cell phenotypes and the rapid regrowth afterward can accelerate the evolution. This process requires a specific time scale to optimally work, as the local death and regrowth is time dependent and for too slow fluctuations the population can be reduced too much to conserve enough variability, so the regrowth is driven by neighboring cells, or too fast that the effect is averaged out as it reaches the lifetime of a cell and merely increases the global death rate.

Although we observe a higher fitness of cells with low adhesion, no clear change in the preferential direction of evolution can be identified along the motility axis.

Our findings indicate an acceleration of the tumor evolution within a surrounding fluctuating nutrient availability when the fluctuations are in the time scale of 15–30 cell generations. Assuming a cell cycle of 24h, the duration of the fluctuation period is on the order of 15–30 days. Therefore, the highly dynamic and unstable nature of initial tumorigenesis could accelerate the adaptation of the tumor composition to its environment.

We predicted that dynamic nutrient availability will introduce a change in fitness of motile cells; this could not be conclusively be answered and has to be explored further by extending the range of possible motility and introducing trade-offs between cellular properties. With the motility implemented here, undisturbed cells perform a true random walk. Different modes of random walk, such as persistent random walks, could induce more collective behavior and therefore change the behavior. Here, we modeled the nutrient fluctuations by moving the nutrient sink in the tumor. Another interesting approach would be to alter this mechanism to move localized sources of nutrients. While this would approximate the situation in an *in vivo* setting with growing and collapsing blood vessels, it would introduce larger areas of low nutrient availability and significantly more cell death. Therefore, this requires larger simulated areas to maintain a sufficient pool of cancer cells to track tumor evolution. Implementing moving nutrient sources in larger simulations is a promising approach for further investigation. Experimental work on spheroid tumors could provide a verification of the frequency dependence of the evolutionary speed, especially the acceleration of tumor evolution on the time scale of 15–30 cell generations. The single cell motility of cells grown in different spheroid cultures could be measured and compared.^26^ The nutrient surrounding of the growing spheroid culture can vary from constant availability to periodic changes in the nutrient concentration in the surrounding medium. We expect to find a faster evolutionary adaption of the composition in the latter setup.

## Data Availability

The simulation code is available as an open source repository: https://gitlab.com/nastja/nastja. The simulation data are available from the authors on reasonable request.

## Supporting Information Available

Configuration file that parametrizes a simulation and an introduction on how to rerun the simulations as well as movies showing the simulations (ZIP)
Description of the software to reproduce the simulations in this study, figure quantifying the lifetime of cells in simulations, figure that compares a threshold based nutrient dependency to a gradual dependency of cell division and death rates on nutrient availability, and figure that indicates the *p*-values for all values shown in Figure 5, as well as the figure only containing early time steps without interaction with the boundaries of parameter space (PDF)
