# Supplementary material for: Multiscale Modeling of Spheroid Tumors: Effect of Nutrient Availability on Tumor Evolution
Source: J Phys Chem B. 2023 Apr 3;127(16):3607–15. doi: 10.1021/acs.jpcb.2c08114 (PMC10150357; doi:10.1021/acs.jpcb.2c08114)
Supplement: Supplementary file 2 — jp2c08114_si_002.pdf [file jp2c08114_si_002.pdf]

Multiscale Modeling of Spheroid Tumors: Effect of Nutrient Availability on Tumor Evolution

Jakob Rosenbauer[1], Marco Berghoff[2], James A. Glazier[3], Alexander Schug[1,4*]

1 Jülich Supercomputing Center, Forschungszentrum Jülich, Jülich, Nordrhein-Westfalen, 52425, Germany
2 Steinbuch Center for Computing, Karlsruhe Institute of Technology, Karlsruhe, Baden-Württemberg, 76021, Germany
3 Biocomplexity Institute, Indiana University, Bloomington, Idiana, 47408, USA
4 University of Duisburg-Essen, Duisburg, Nordrhein-Westfalen, 47057, Germany

* al.schug@fz-juelich.de

Supporting Information for publication

Reproduce the simulations:
In order to reproduce the simulation results , first install the NAStJA framework, we have compiled a tutorial on the installation here: https://nastja.gitlab.io/nastja/docs/set_up.html Once the software is installed and tested a simulation can be started with the attached configuration file by running:

```
mpirun -n 48 nastja -c SimulationConfig.json -o out0
```

This will run the simulation and write the output files into the folder out0. Those simulation outputs can be analyzed with out python tool nastjapy (https://gitlab.com/nastja/nastjapy). In order to vary the period T in the simulations the parameter
$"CellsInSilico" : "signaling" : "wobblet" : [100, 100, 0]$ has to be altered in the simulation configuration file, here the vector specifies the period time for each spatial dimension.

Lifetime of cells

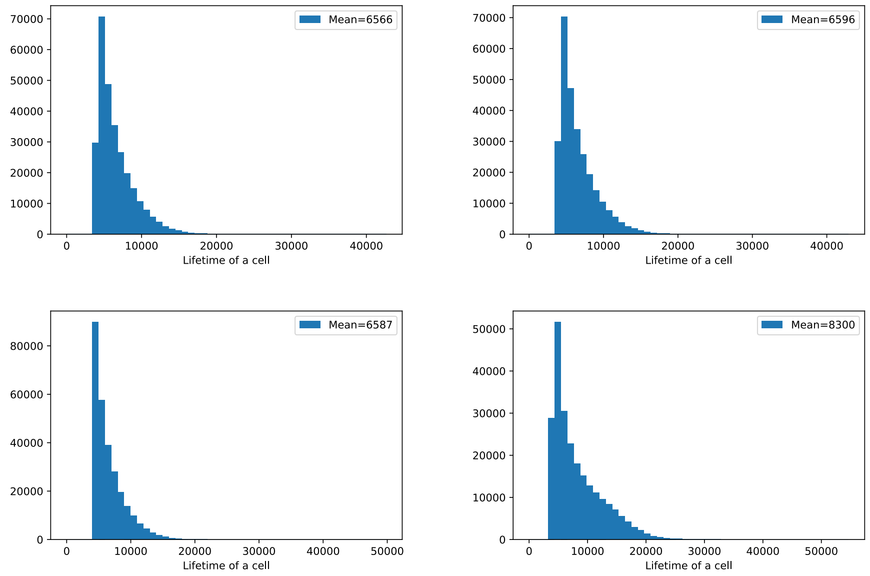

Fig. S1. Histograms of the lifetime of cells of four simulations

Threshold-based nutrient dependency: Additionally we tested a threshold-based dependency of cell division and death, whic introduces a constant division probability if a cell exceeds a certain nutrient value and a step decreases the death rate above a second threshold. This significantly decreases the evolutionary speed of the system (see Fig S2), furthermore, a linear dependency is biologically more reasonable, since cells do not binarily up- or down-regulate cell division in most cases, but adapt continuously. Hence, we introduce a linear rate dependency which is used in the main text of the manuscript.

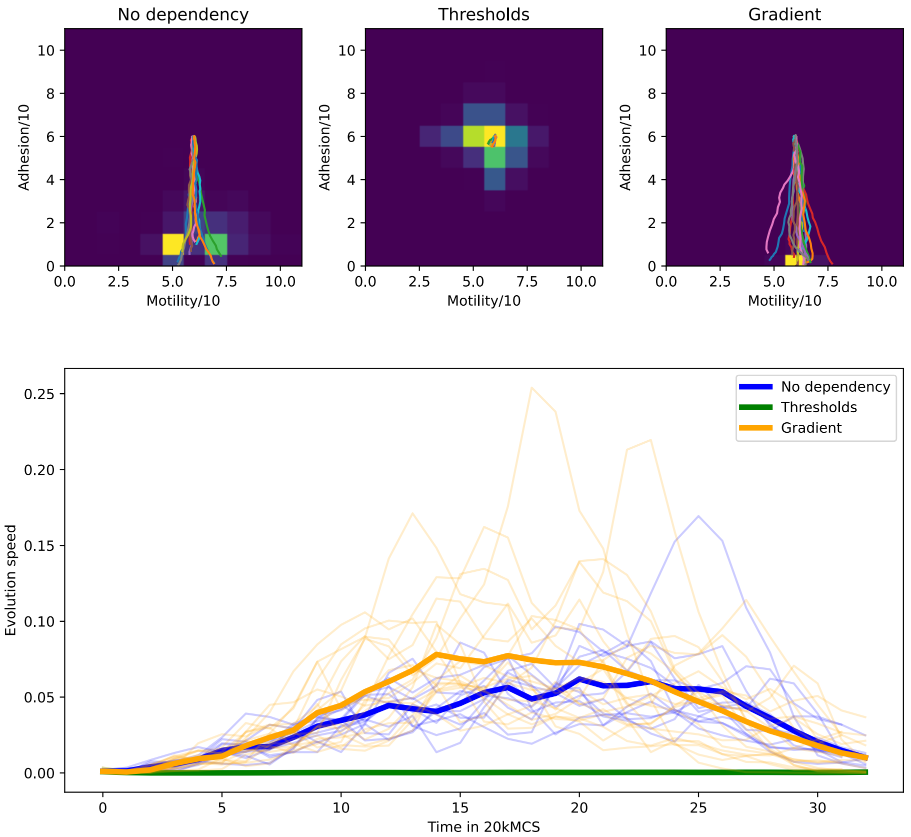

Fig. S2. Spheroidal tumor growth for different nutrient dependency mechanisms, including threshold based dependency. The top plots show the temporal trajectories of the centroid of the phase space occupation, originating in the center and developing towards low adhesion. The shading shows the distribution in the phenotype space at the endpoint at $t = 580\,\mathrm{kMCS}$ of a single simulation. The bottom plot shows the evolution speed of different iterations over time, as well as the mean evolution speed of this case. Three different cased of dependencies of the division and death rates of single cells are compared: no dependency of cell division and death on nutrient availability, division and death binarily turned on and off by thresholds on the nutrient availability and linear dependence of the rates on the nutrient availability.

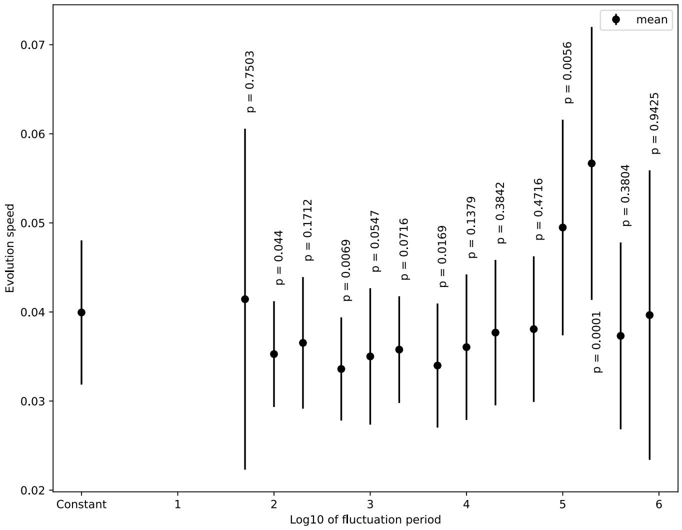

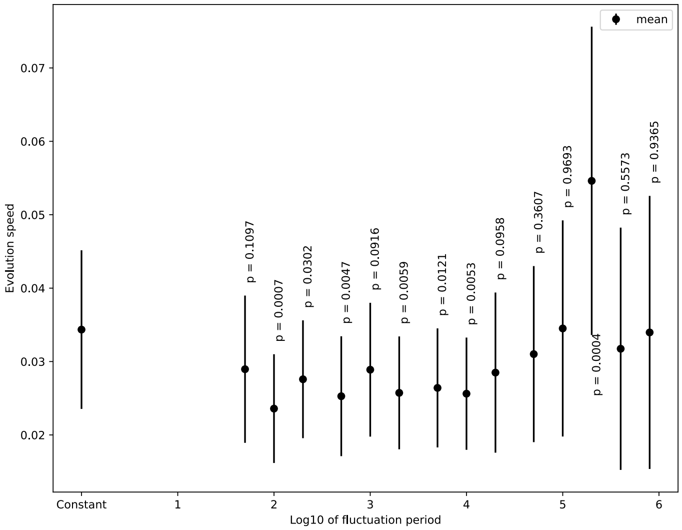

Fig. S3. Reproductions of main text Fig. 5 b. **a**: All p-values of the t-test comparing the 'constant' case (no movement in the nutrient dip) with the dynamic cases of indicated fluctuation periods. **b**: Here, only times with T<340kMCS were used to obtain the speed of the evolution. This ensures no interaction of the parameter distribution of the cell types with the bounds of the parameter range (cf. main text Fig. 4 b+c). While reproducing the main results the statistics are decreased. Therefore, the full length of the simulations are used in the main text.
